# Homogeneity score test of AC_1_ statistics and estimation of common AC_1_ in multiple or stratified inter-rater agreement studies

**DOI:** 10.1186/s12874-019-0887-5

**Published:** 2020-02-05

**Authors:** Chikara Honda, Tetsuji Ohyama

**Affiliations:** 1 Oncology Research Center, Ono Pharmaceutical Co., Ltd., 3-1-1 Sakurai, Mishima-gun, Osaka, 618-8585 Japan; 2 Graduate School of Medicine, Kurume University, 67 Asahi-machi, Kurume, Fukuoka, 830-0011 Japan; 3 Biostatistics Center, Kurume University, 67 Asahi-machi, Kurume, Fukuoka, 830-0011 Japan

**Keywords:** Common AC_1_, Consistency evaluation, Gwet’s AC_1_, Homogeneity test, Inter-rater agreement, Stratified study

## Abstract

**Background:**

Cohen’s *κ* coefficient is often used as an index to measure the agreement of inter-rater determinations. However, *κ* varies greatly depending on the marginal distribution of the target population and overestimates the probability of agreement occurring by chance. To overcome these limitations, an alternative and more stable agreement coefficient was proposed, referred to as Gwet’s AC_1_. When it is desired to combine results from multiple agreement studies, such as in a meta-analysis, or to perform stratified analysis with subject covariates that affect agreement, it is of interest to compare several agreement coefficients and present a common agreement index. A homogeneity test of *κ* was developed; however, there are no reports on homogeneity tests for AC_1_ or on an estimator of common AC_1_. In this article, a homogeneity score test for AC_1_ is therefore derived, in the case of two raters with binary outcomes from K independent strata and its performance is investigated. An estimation of the common AC_1_ between strata and its confidence intervals is also discussed.

**Methods:**

Two homogeneity tests are provided: a score test and a goodness-of-fit test. In this study, the confidence intervals are derived by asymptotic, Fisher’s Z transformation and profile variance methods. Monte Carlo simulation studies were conducted to examine the validity of the proposed methods. An example using clinical data is also provided.

**Results:**

Type I error rates of the proposed score test were close to the nominal level when conducting simulations with small and moderate sample sizes. The confidence intervals based on Fisher’s Z transformation and the profile variance method provided coverage levels close to nominal over a wide range of parameter combination.

**Conclusions:**

The method proposed in this study is considered to be useful for summarizing evaluations of consistency performed in multiple or stratified inter-rater agreement studies, for meta-analysis of reports from multiple groups and for stratified analysis.

## Background

To evaluate the reliability when two raters classify objects as either positive (+) or negative (−), Cohen’s *κ* [1] and the intra-class version of *κ*, which is identical to Scott’s *π* [2], have often been used. Let *p*_*a*_ be the agreement probability, and *p*_1_ and *p*_2_ the probabilities classified as (+) by rater 1 and 2 respectively. Then Cohen’s *κ* (*κ*_*Cohen*_) and Scott’s *π* (*κ*_*Scott*_) are defined as follows:

$$ {\kappa}_{Cohen}=\frac{p_a-{p}_{e(c)}}{1-{p}_{e(c)}},\kern0.5em {\kappa}_{Scott}=\frac{p_a-{p}_{e(s)}}{1-{p}_{e(s)}}, $$

where ${p}_{e(c)}={p}_1{p}_2+\left(1-{p}_1\right)\left(1-{p}_2\right),{p}_{e(s)}={p}_{+}^2+{\left(1-{p}_{+}\right)}^2$ and *p*_+_ = (*p*_1_ + *p*_2_)/2. The *p*_*e*(*c*)_ and *p*_*e*(*s*)_ are the probabilities of agreement expected by chance for Cohen’s *κ* and Scott’s *π* respectively. The *p*_*e*(*c*)_ assumes that the probabilities of positive classification differ between two raters, while the *p*_*e*(*s*)_ assumes that these two probabilities are the same. Landis and Koch provided benchmarks of the strength of consistency as follows: values ≤0 as poor, 0.00 to 0.20 as slight, 0.21 to 0.40 as fair, 0.41 to 0.60 as moderate, 0.61 to 0.80 as substantial and 0.81 to 1.00 as almost perfect agreement [3]. Although the authors acknowledge the arbitrary nature of their benchmarks, they recommended their benchmark scale as a useful guideline for practitioners.

Many extensions have been made to Cohen’s *κ* including those for agreement in the cases of ordinal data [4], multiple raters [5–9], comparisons of correlated *κ* ’s [10–13] and stratified data [14, 15]. However, as Feinstein and Cicchetti showed, Cohen’s *κ* depends strongly on the marginal distributions and therefore behaves paradoxically [16]. This behavior can be explained by the bias effect and the prevalence effect, on which various discussions have been undertaken [16–18]. A number of alternative measures of agreements have also been proposed, such as Holley and Guilford’s G [19], Aickin’s *α* [20], Andres and Marzo’s delta [21], Marasini’s s* [22, 23] and Gwet’s AC_1_ [24] and AC_2_ [25].

Gwet showed that AC_1_ has better statistical properties (bias and variance) than Cohen’s *κ*, Scott’s *π* and G-index under a limited set of simulations for two raters with binary outcomes [24]. Shanker and Bangdiwala compared Cohen’s *κ*, Scott’s *π*, Prevalence Adjusted Bias Adjusted Kappa (PABAK) [26], AC_1_ and B-statistic [27], which is not a kappa-type chance-corrected measure, in the case of two raters and binary outcomes and showed that AC_1_ has better properties than other kappa-type measures [28]. In addition, AC_1_ has been utilized in the field of medical research over the past decade [29–35]. Therefore, in this study we have limited our discussion to AC_1_ in the case of two raters with binary outcomes.

First, a brief review of the concept of Gwet’s AC_1_ is provided. Consider the situation in which two raters independently classify randomly extracted subject as positive (+) or negative (−). Gwet defined two events: G = {the two raters agree} and R = {at least one rater performs random rating}. The probability of agreement expected by chance is then *p*_*e*_ = *P*(*G* ⋂ *R*) = *P*(*G*| *R*)*P*(*R*). A random rating would lead to the classification of an individual into each category with the same probability $\frac{1}{2}$ and it follows that $P\left(G|R\right)=2\times \left(\frac{1}{2}\right)\times \left(\frac{1}{2}\right)=\frac{1}{2}$. As for the estimation of *P*(*R*), this probability cannot be obtained from data. Therefore, Gwet proposed approximating it with a normalized measure of randomness Ψ, defined as follows:

1 $$ P(R)\simeq \Psi =\frac{\pi_{+}\left(1-{\pi}_{+}\right)}{\frac{1}{2}\left(1-\frac{1}{2}\right)}=4{\pi}_{+}\left(1-{\pi}_{+}\right), $$

where *π*_+_ is the probability that a randomly chosen rater classifies a randomly chosen subject into the + category. Thus, the approximated probability of chance agreement is represented by

2 $$ {p}_e^{\ast }=P\left(G|R\right)\Psi =2{\pi}_{+}\left(1-{\pi}_{+}\right). $$

AC_1_ is thus defined as follows:

3 $$ \gamma =\frac{p_a-{p}_e^{\ast }}{1-{p}_e^{\ast }}, $$

where *p*_*a*_ is the probability of agreement. Although *p*_*e*_ is approximated to ${p}_e^{\ast }$, Gwet showed that the bias of *γ*, the difference between *γ* and the true inter-rater reliability, is equal to or less than Cohen’s *κ*, Scott’s *π* and G-index under some assumption in the case of two raters with binary outcomes. Gwet also provided an estimator ${\hat{\gamma}}^{\ast }$ of *γ* and its variance for multiple raters and multiple categories based on the randomization approach, which requires the selection of subjects to be random in such a way that all possible subject samples have the exact same chance of being selected. However, it is advantageous to employ a model-based approach when, for example, the evaluation of the effect of subject covariates on agreement is of interest. Therefore, in the case of two raters with binary outcomes, Ohyama [36] assumed the underlying probability that a subject is rated as (+) and its marginal homogeneity of the two raters, and then constructed the likelihood. The maximum likelihood estimator of *γ*, which is shown to be identical to the estimator given by Gwet, was derived. The likelihood-based confidence intervals for AC_1_, inclusion of subject covariates, hypothesis testing and sample size determination were also discussed [36].

In this article, we discuss stratification analyses as another approach to adjust the effect of subject covariates on agreement. For example, a clinical assessment whether a patient has a particular disease symptom may be influenced by overall severity of the disease. In such a case, we consider stratification based on the severity of the disease. Another example is a multicenter inter-rater agreement study, in which the classifications for subjects are conducted independently in each center. These situations require several independent agreement statistics. Then the main purpose of the analyses would be testing whether the degree of inter-rater agreement can be regarded as homogeneous across strata, such as centers and severities of the disease.

For *κ*, Fleiss has been at the forefront of the idea of *χ*^2^ test-based inter-class consistency with large sample variances [37] and further studies by Donner, Eliasziw and Klar [14], Nam [15, 38] and Wilding, Consiglio and Shan [39] have developed the homogeneity test of *κ* across covariate levels. However, there are no reports on homogeneity tests for AC_1_ or on an estimator of common AC_1_. Therefore, in this article, we derive the homogeneity score test for AC_1_ from *K* independent strata and its performance is investigated. An estimation of the common AC_1_ between strata and its confidence intervals is also discussed. Finally, an example application of our approach to clinical trial data is provided.

## Methods

### Homogeneity tests

#### Score test

Consider *K* independent strata involving *n*_*k*_ subjects for *k* = 1, …, *K*. In each stratum, two raters independently classify subjects as either positive (+) or negative (−). Let *X*_*kij*_= 1 if subject *i*(=1, …, *n*_*k*_) in the *k-*th stratum is classified as “+” by rater *j*(=1, 2) and *X*_*kij*_ = 0 otherwise. Suppose that *P*(*X*_*kij*_ = 1| *i*) = *u*_*ki*_, $E\left({u}_{ki}\right)={\pi}_k\ \mathrm{and}\kern0.5em Var\left({u}_{ki}\right)={\sigma}_k^2$. The *γ* of the *k*-th stratum is then expressed as follows [36]:

4 $$ {\gamma}_k=\frac{1+2\left[{\sigma_k}^2-2{\pi}_k\left(1-{\pi}_k\right)\right]}{1-2{\pi}_k\left(1-{\pi}_k\right)}. $$

Let the number of observed pairs in the three categories of the *k*-th stratum be *x*_1*k*_, *x*_2*k*_ and *x*_3*k*_ and their corresponding probabilities be *P*_1*k*_(*γ*_*k*_), *P*_2*k*_(*γ*_*k*_) and *P*_3*k*_(*γ*_*k*_). The data of the *k*-th stratum are then given as shown in Table 1.

**Table 1:** Data layout

Category	Ratings	Frequency	Probability
1	(+, +)	*x*_1*k*_	*P*_1*k*_(*γ*_*k*_)
2	(+, −) or (−, +)	*x*_2*k*_	*P*_2*k*_(*γ*_*k*_)
3	(−, −)	*x*_3*k*_	*P*_3*k*_(*γ*_*k*_)
Total		*n*_*k*_	1

The log-likelihood function is given by

5 $$ l\left(\boldsymbol{\gamma}, \boldsymbol{\pi} \right)=\sum \limits_{k=1}^K{l}_k\left({\gamma}_k,{\pi}_k\right), $$

where **γ** = (*γ*_1_, …, *γ*_*K*_)^'^, **π** = (*π*_1_, …, *π*_*K*_)^'^, *l*_*k*_(*γ*_*k*_, *π*_*k*_) = *x*_1*k*_ log *P*_1*k*_(*γ*_*k*_) + *x*_2*k*_ log *P*_2*k*_(*γ*_*k*_) + *x*_3*k*_ log *P*_3*k*_(*γ*_*k*_),

6 $$ {P}_{1k}\left({\gamma}_k\right)={\pi}_k\left(2-{\pi}_k\right)-\frac{1}{2}+\frac{\gamma_k}{2}{A}_k, $$

7 $$ {P}_{2k}\left({\gamma}_k\right)={A}_k\left(1-{\gamma}_k\right), $$

8 $$ {P}_{3k}\left({\gamma}_k\right)=\left(1-{\pi}_k\right)\left(1+{\pi}_k\right)-\frac{1}{2}+\frac{\gamma_k}{2}{A}_k, $$

and *A*_*k*_ = 1 − 2*π*_*k*_(1 − *π*_*k*_).

The maximum likelihood estimators of *γ*_*k*_ and *π*_*k*_ are then given by

9 $$ {\hat{\gamma}}_k=1-\frac{2{n}_k{x}_{2k}}{n_k^2+{\left({x}_{1k}-{x}_{3k}\right)}^2} $$

and

10 $$ {\hat{\pi}}_k=\frac{2{x}_{1k}+{x}_{2k}}{2{n}_k}, $$

respectively.

The first and second derivatives of the log-likelihood function and the Fisher information matrix are given in the Appendix. The aim of this study is to test the homogeneity of the agreement coefficients among *K* strata, and thus the null hypothesis to test is represented by *H*_0_ : *γ*_*k*_ = *γ*_0_ (*k* = 1, 2, ..., *K*). The score test statistic for the null hypothesis is derived as follows (see Appendix):

11 $$ T\left({\overset{\sim }{\gamma}}_0,\overset{\sim }{\boldsymbol{\pi}}\right)=\sum \limits_{k=1}^K\frac{{{\overset{\sim }{R}}_k}^2{\overset{\sim }{D}}_k}{n_k\left({\overset{\sim }{B}}_k{\overset{\sim }{D}}_k-{{\overset{\sim }{C}}_k}^2\right)}, $$

where ${\overset{\sim }{B}}_k,{\overset{\sim }{C}}_k,{\overset{\sim }{D}}_k\ \mathrm{and}\ {\overset{\sim }{R}}_k$ are obtained by substituting the maximum likelihood estimators ${\overset{\sim }{\gamma}}_0$ and ${\overset{\sim }{\pi}}_k$ under the null hypothesis into $$ {\displaystyle \begin{array}{l}{B}_k=\frac{1}{P_{1k}}+\frac{4}{P_{2k}}+\frac{1}{P_{3k}},\\ {}{C}_k=\frac{1}{P_{1k}}-\frac{1}{P_{3k}}+\left(1-{\gamma}_k\right)\left(1-2{\pi}_k\right){B}_k,\\ {}{D}_k=\frac{1}{P_{1k}}+\frac{1}{P_{3k}}+\left(1-{\gamma}_k\right)\left(1-2{\pi}_k\right)\left(\frac{1}{P_{1k}}-\frac{1}{P_{3k}}+{C}_k\right),\\ {}{R}_k=\frac{x_{1k}}{P_{1k}}-\frac{2{x}_{2k}}{P_{2k}}+\frac{x_{3k}}{P_{3k}}.\end{array}} $$

$T\left({\overset{\sim }{\gamma}}_0,\overset{\sim }{\boldsymbol{\pi}}\right)$ is asymptotically distributed as a *χ*^2^ with *K* − 1 degrees of freedom. The homogeneity hypothesis is rejected at level *α* when $T\left({\overset{\sim }{\gamma}}_0,\overset{\sim }{\boldsymbol{\pi}}\right)$ ≥ $\kern0.5em {\chi}_{\left(1-\alpha \right),K-1}^2$, where ${\chi}_{\left(1-\alpha \right),K-1}^2$ is the 100 × (1 − *α*) percentile point of the *χ*^2^ distribution with *K* − 1 degrees of freedom.

Note that, since 0 ≤ *P*_1*k*_(*γ*_*k*_), *P*_2*k*_(*γ*_*k*_), *P*_3*k*_(*γ*_*k*_) ≤ 1 and *P*_1*k*_(*γ*_*k*_) + *P*_2*k*_(*γ*_*k*_) + *P*_3*k*_(*γ*_*k*_) = 1, substituting (6), (7) and (8) into these equations, the admissible range of *γ*_*k*_ with respect to *π*_*k*_ is obtained as follows [36]:

12 $$ \frac{2-\left(1-|1-2{\pi}_k|\right)\left(3+|1-2{\pi}_k|\right)}{2-\left(1-|1-2{\pi}_k|\right)\left(1+|1-2{\pi}_k|\right)}\le {\gamma}_k\le 1. $$

When obtaining the maximum likelihood estimators ${\overset{\sim }{\gamma}}_0$ and ${\overset{\sim }{\pi}}_k$ under the null hypothesis by numerical calculation, initial values need to be set to satisfy this condition.

#### Goodness-of-fit test

Donner, Eliasziw and Klar proposed a goodness-of-fit approach for testing homogeneity of kappa statistics in the case of two raters with binary outcomes [40]. This procedure can also be applied to AC_1_ statistics. Given that the frequencies *x*_1*k*_, *x*_2*k*_, *x*_3*k*_, *k* = 1, …, *K* in Table 1 follow a multinomial distribution conditional on *n*_*k*_, estimated probabilities under *H*_0_ are given by ${\hat{P}}_{hk}\left({\overset{\sim }{\gamma}}_0\right)$, which is obtained by replacing *π*_*k*_ by ${\hat{\pi}}_k$ and *γ*_*k*_ by ${\overset{\sim }{\gamma}}_0$ in *P*_*hk*_(*γ*_*k*_); *h* = 1, 2, 3; *k* = 1, …, *K*. Then the goodness-of-fit statistic is derived as follows:

13 $$ {\chi}_G^2=\sum \limits_{k=1}^K\sum \limits_{h=1}^3\frac{{\left({x}_{hk}-{n}_k{\hat{P}}_{hk}\left({\tilde{\gamma}}_0\right)\right)}^2}{n_k{\hat{P}}_{hk}\left({\tilde{\gamma}}_0\right)}, $$

under *H*_0_, ${\chi}_G^2$ follows an approximate *χ*^2^ distribution with *K* − 1 degrees of freedom. The homogeneity hypothesis is rejected at level *α* when ${\chi}_G^2\ge {\chi}_{\left(1-\alpha \right),K-1}^2$, where ${\chi}_{\left(1-\alpha \right),K-1}^2$ is the 100 × (1 − *α*) percentile point of the *χ*^2^ distribution with *K* − 1 degrees of freedom.

### Estimation of common AC_1_

If the assumption of homogeneity is reasonable, the estimate of *γ*_0_ can be used as an appropriate summary measure of reliability. The maximum likelihood estimator ${\overset{\sim }{\gamma}}_0$ and ${\overset{\sim }{\pi}}_k$ are obtained by maximizing the log-likelihood functions ${l}_0\left({\gamma}_0,\boldsymbol{\pi} \right)=\sum \limits_{k=1}^K{l}_k\left({\gamma}_0,{\pi}_k\right)$. Since an analytical solution cannot be obtained from this function, numerical iterative calculations are used. The variance $Var\left({\overset{\sim }{\gamma}}_0\right)$ of ${\overset{\sim }{\gamma}}_0$ can be expressed as follows (see Appendix):

14 $$ Var\left({\tilde{\gamma}}_0\right)=4{\left[\sum \limits_{k=1}^K{n}_k{A_k}^2\left({B}_k^{(0)}-\frac{{C_k^{(0)}}^2}{D_k^{(0)}}\right)\right]}^{-1}={\left[\sum \limits_{k=1}^K\frac{1}{Va{r}_k\left({\tilde{\gamma}}_0\right)}\right]}^{-1}, $$

where ${B}_k^{(0)},{C}_k^{(0)},{D}_k^{(0)}$ are values using *γ*_*k*_ = *γ*_0_ in *B*_*k*_, *C*_*k*_, *D*_*k*_ respectively, and

15 $$ Va{r}_k\left({\tilde{\gamma}}_0\right)=\frac{1}{n_k{A}_k^2}\left[{A}_k\left(1-{\gamma}_0\right)-\left({A}_k^2-4{A}_k+2\right){\left(1-{\gamma}_0\right)}^2-{A}_k\left(2{A}_k-1\right){\left(1-{\gamma}_0\right)}^3\right]. $$

A simple 100 × (1 − *α*) % confidence interval using the asymptotic normality of ${\overset{\sim }{\gamma}}_0$ can be expressed as follows:

16 $$ {\tilde{\gamma}}_0\pm {Z}_{\raisebox{1ex}{$\alpha $}\!\left/ \!\raisebox{-1ex}{$2$}\right.}\sqrt{\hat{Var}\left({\tilde{\gamma}}_0\right)}, $$

where *Z*_*α*/2_ is the *α*/2 upper quantile of the standard normal distribution and $\hat{Var}\left({\overset{\sim }{\gamma}}_0\right)$ is obtained by substituting ${\overset{\sim }{\gamma}}_0$ and ${\overset{\sim }{\pi}}_k$ into (14). Hereafter, this method is referred to as the simple asymptotic (SA) method. Since Eq. (14) depends on *γ*_0_, SA method may not have the correct coverage rate, and the normality of the sampling distribution of ${\overset{\sim }{\gamma}}_0$ may be improved using Fisher’s Z transformation. This method is referred to below as Fisher’s Z transformation (FZ) method (see Appendix).

As an alternative method, we employ the profile variance approach, which has been shown to perform well in the case of the intra-class *κ* for binary outcome data [41–43]. This approach also performs well for AC_1_ in the case of two raters with binary outcomes [36]. The confidence interval based on the profile variance can be obtained by solving the following inequality for *γ*_0_:

17 $$ \frac{{\left(\overset{\sim }{\boldsymbol{\gamma}}-{\gamma}_0\right)}^2}{\overset{\sim }{Var}\left({\overset{\sim }{\gamma}}_0\right)}\le {Z}_{\mathrm{a}/2}^2, $$

where $\overset{\sim }{Var}\left({\overset{\sim }{\gamma}}_0\right)$ is given by substituting ${\overset{\sim }{\pi}}_k$ into *π*_*k*_ in (15). Hereafter, this method is referred to as the profile variance (PV) method (see Appendix).

### Numerical evaluations

We conducted Monte Carlo simulations to investigate the performance of the proposed homogeneity tests and to evaluate the estimate of common AC_1_ and its confidence intervals under the following conditions: the number of strata in the simulation is *K* = 2 or 3; and random observations are generated from the trinomial distributions according to the probabilities of (6), (7) and (8) by giving the values of *γ*_*k*_ and *π*_*k*_. The balanced and unbalanced cases were considered for the values of *π*_*k*_ and *n*_*k*_. The values of *γ*_*k*_ and *π*_*k*_ are set within the theoretical range of Eq. (12) derived in the preceding paragraph. Ten thousand times of iterations were carried out for each parameter combination.

When *π*_*k*_ is close to 0 or 1 and *n*_*k*_ is small, there are cases in which the generated data include zero cells. In such cases, *B*_*k*_, *C*_*k*_, *D*_*k*_ and *R*_*k*_ cannot be estimated . Thus, when zero cells were generated, we adopted the approach of adding 0.5 to the frequency of each combination by two raters, (+,+), (+,−), (−,+), (−,−). This simple method was discussed by Agresti [44] and was adopted in a previous study [39].

## Results

### Empirical type I error rate for the homogeneity test

The type I error rates of the homogeneity tests with a significance level of 0.05 were examined. The sample size was set at *n*_*k*_ = *n* = 20, 50, 80 for balanced settings and (*n*_1_, *n*_2_, *n*_3_) = (20, 50, 80) for unbalanced settings. The error rate obtained by the score test is expressed as SCORE and the error rate obtained by the goodness-of-fit test is expressed as GOF. Table 2 summarizes the results for *K* = 2.

**Table 2:** Empirical type I error rates of homogeneity tests for *γ*_1_ = *γ*_2_ = *γ*_0_ based on 10,000 simulations (*K =* 2 balanced sample size)

Balanced *π* conditions					Unbalanced *π* conditions					
*n*_1_ = *n*_2_	*γ*_0_	*π*_1_ = *π*_2_	SCORE	GOF	*n*_1_ = *n*_2_	*γ*_0_	π_1_	π_2_	SCORE	GOF
20	0.1	0.5	0.045	0.067	20	0.1	0.5	0.35	0.049	0.097
	0.3		0.046	0.067		0.3			0.049	0.096
	0.5		0.048	0.062		0.5			0.050	0.083
	0.7		0.033	0.041		0.7			0.037	0.049
	0.9		0.002	0.003		0.9			0.003	0.005
	0.1	0.35	0.052	0.121		0.1	0.65	0.35	0.050	0.120
	0.3		0.054	0.126		0.3			0.051	0.120
	0.5		0.052	0.103		0.5			0.051	0.101
	0.7		0.039	0.064		0.7			0.039	0.065
	0.9		0.004	0.006		0.9			0.004	0.007
	0.7	0.2	0.047	0.132		0.7	0.5	0.2	0.038	0.090
	0.9		0.008	0.029		0.9			0.005	0.013
50	0.1	0.5	0.050	0.058	50	0.1	0.5	0.35	0.048	0.117
	0.3		0.047	0.054		0.3			0.051	0.087
	0.5		0.050	0.054		0.5			0.049	0.072
	0.7		0.051	0.053		0.7			0.050	0.060
	0.9		0.026	0.027		0.9			0.024	0.027
	0.1	0.35	0.051	0.172		0.1	0.65	0.35	0.051	0.168
	0.3		0.051	0.126		0.3			0.049	0.117
	0.5		0.052	0.092		0.5			0.051	0.092
	0.7		0.052	0.072		0.7			0.052	0.071
	0.9		0.028	0.033		0.9			0.028	0.033
	0.7	0.2	0.053	0.162		0.7	0.5	0.2	0.051	0.104
	0.9		0.037	0.061		0.9			0.032	0.042
80	0.1	0.5	0.047	0.052	80	0.1	0.5	0.35	0.051	0.120
	0.3		0.047	0.051		0.3			0.053	0.094
	0.5		0.054	0.057		0.5			0.051	0.072
	0.7		0.050	0.052		0.7			0.051	0.061
	0.9		0.037	0.039		0.9			0.047	0.050
	0.1	0.35	0.052	0.173		0.1	0.65	0.35	0.052	0.172
	0.3		0.054	0.123		0.3			0.054	0.124
	0.5		0.053	0.089		0.5			0.055	0.090
	0.7		0.051	0.069		0.7			0.051	0.069
	0.9		0.044	0.051		0.9			0.045	0.052
	0.7	0.2	0.052	0.152		0.7	0.5	0.2	0.050	0.103
	0.9		0.051	0.073		0.9			0.048	0.059

Overall, the proposed score test did not show any significant type I error rate inflation, but it was very conservative when sample size was small and *γ*_0_ was close to 1.

In the case of *n* = 20 when *γ*_0_ = 0.1, 0.3 or 0.5, the type I error rates of SCORE were maintained at the nominal level of 0.05 regardless of whether *π*_*k*_ was balanced or unbalanced, but when *γ*_0_ = 0.7 or 0.9, the type I error rates were slightly conservative. Especially when *γ*_0_ = 0.9, the rate was significantly conservative to the extent of being less than 0.01. In the case of *n* = 50, the type I error rates were maintained at the nominal level of 0.05 except when *γ*_0_ = 0.9. Finally in the case of *n* = 80, the type I error rates were almost maintained at the nominal level. In contrast, the type I error rate of GOF tended to be larger than that of SCORE and in many cases it was not maintained at the nominal level.

The results obtained for *K* = 3 are shown Table S1 and Table S2 in Additional file 1.

The Additional file 2 provides the simulation code of empirical type I error rate using R language.

### Empirical power of the homogeneity test

The empirical power of the score test was investigated only for the case of *K* = 2, by setting *γ*_1_ = 0.1, 0.3, 0.5 and *γ*_2_ − *γ*_1_ = 0.3, 0.4. The values of *π*_*k*_ and *n*_*k*_ were set as in the type I error simulation. The results are shown in Table 3. The power tended to be large as the value of *γ*_1_ increased under the fixed values of *π* and *γ*_2_ − *γ*_1_.

**Table 3:** Empirical power of homogeneity tests based on 10,000 simulations (*K* = 2 balanced sample size)

Balanced *π* conditions						Unbalanced *π* conditions						
*n*_1_ = *n*_2_	*γ*_1_	*γ*_2_	*π*_1_ = *π*_2_	SCORE	GOF	*n*_1_ = *n*_2_	*γ*_1_	*γ*_2_	*π*_1_	*π*_2_	SCORE	GOF
20	0.1	0.5	0.5	0.243	0.290	20	0.1	0.5	0.5	0.35	0.234	0.304
	0.3	0.6		0.173	0.202		0.3	0.6			0.168	0.224
	0.3	0.7		0.294	0.323		0.3	0.7			0.293	0.351
	0.5	0.8		0.212	0.232		0.5	0.8			0.216	0.258
	0.5	0.9		0.372	0.396		0.5	0.9			0.389	0.430
	0.1	0.5	0.35	0.245	0.357		0.1	0.5	0.65	0.35	0.243	0.355
	0.3	0.6		0.185	0.279		0.3	0.6			0.171	0.266
	0.3	0.7		0.313	0.408		0.3	0.7			0.296	0.398
	0.5	0.8		0.227	0.295		0.5	0.8			0.221	0.291
	0.5	0.9		0.396	0.483		0.5	0.9			0.394	0.475
	0.5	0.8	0.2	0.270	0.411		0.5	0.8	0.5	0.2	0.230	0.278
	0.5	0.9		0.482	0.616		0.5	0.9			0.435	0.473
50	0.1	0.5	0.5	0.538	0.562	50	0.1	0.5	0.5	0.35	0.525	0.595
	0.3	0.6		0.377	0.396		0.3	0.6			0.369	0.428
	0.3	0.7		0.635	0.651		0.3	0.7			0.630	0.673
	0.5	0.8		0.512	0.525		0.5	0.8			0.517	0.548
	0.5	0.9		0.835	0.841		0.5	0.9			0.843	0.855
	0.1	0.5	0.35	0.509	0.652		0.1	0.5	0.65	0.35	0.503	0.648
	0.3	0.6		0.379	0.485		0.3	0.6			0.364	0.470
	0.3	0.7		0.633	0.718		0.3	0.7			0.618	0.711
	0.5	0.8		0.518	0.585		0.5	0.8			0.516	0.581
	0.5	0.9		0.844	0.877		0.5	0.9			0.838	0.874
	0.5	0.8	0.2	0.552	0.711		0.5	0.8	0.5	0.2	0.531	0.598
	0.5	0.9		0.878	0.915		0.5	0.9			0.861	0.863
80	0.1	0.5	0.5	0.757	0.768	80	0.1	0.5	0.5	0.35	0.732	0.786
	0.3	0.6		0.568	0.578		0.3	0.6			0.545	0.596
	0.3	0.7		0.841	0.847		0.3	0.7			0.833	0.858
	0.5	0.8		0.716	0.722		0.5	0.8			0.717	0.741
	0.5	0.9		0.967	0.968		0.5	0.9			0.966	0.970
	0.1	0.5	0.35	0.707	0.819		0.1	0.5	0.65	0.35	0.707	0.816
	0.3	0.6		0.538	0.645		0.3	0.6			0.541	0.644
	0.3	0.7		0.826	0.879		0.3	0.7			0.822	0.884
	0.5	0.8		0.717	0.767		0.5	0.8			0.715	0.764
	0.5	0.9		0.963	0.973		0.5	0.9			0.965	0.974
	0.5	0.8	0.2	0.746	0.872		0.5	0.8	0.5	0.2	0.734	0.787
	0.5	0.9		0.976	0.982		0.5	0.9			0.974	0.970

The empirical power of the GOF test was also examined under the same simulation conditions as the score test. The results are also shown in Table 3. However, the GOF had a large type I error rate inflation (Table 2) and was invalid as a test.

The Additional file 2 provides the simulation code of empirical power using R language.

### Bias and mean square error for common AC_1_

We evaluated the bias and mean square error (MSE) of the maximum likelihood estimator for the common AC_1_, ${\overset{\sim }{\gamma}}_0$. The balanced and unbalanced conditions for *π*_*k*_ and the balanced condition for *n*_*k*_ were considered. The results are shown in Table 4. The bias of ${\overset{\sim }{\gamma}}_0$ tended to be small as *γ*_0_ increased, but ${\overset{\sim }{\gamma}}_0$ was almost unbiased. As expected, the bias and MSE tended to be small as the sample size increased.

**Table 4:** Bias and mean square error of the maximum likelihood estimator for the common AC_1_ based on 10,000 simulations (*K* = 2 balanced sample size)

Balanced *π* conditions					Unbalanced *π* conditions					
*n*_1_ = *n*_2_	*γ*_0_	*π*_1_ = *π*_2_	Bias	MSE	*n*_1_ = *n*_2_	*γ*_0_	*π*_1_	*π*_2_	Bias	MSE
20	0.1	0.5	0.026	0.025	20	0.1	0.5	0.35	0.019	0.027
	0.3		0.023	0.023		0.3			0.018	0.024
	0.5		0.017	0.018		0.5			0.013	0.019
	0.7		0.009	0.012		0.7			0.007	0.012
	0.9		−0.011	0.003		0.9			−0.011	0.003
	0.1	0.35	0.009	0.029		0.1	0.65	0.35	0.010	0.028
	0.3		0.007	0.025		0.3			0.011	0.025
	0.5		0.007	0.019		0.5			0.008	0.019
	0.7		0.004	0.012		0.7			0.004	0.012
	0.9		−0.011	0.003		0.9			−0.011	0.003
	0.7	0.2	−0.007	0.012		0.7	0.5	0.2	0.001	0.012
	0.9		−0.010	0.003		0.9			−0.010	0.003
80	0.1	0.5	0.007	0.006	80	0.1	0.5	0.35	0.006	0.007
	0.3		0.006	0.006		0.3			0.005	0.006
	0.5		0.005	0.005		0.5			0.004	0.005
	0.7		0.003	0.003		0.7			0.003	0.003
	0.9		0.002	0.001		0.9			0.001	0.001
	0.1	0.35	0.004	0.007		0.1	0.65	0.35	0.003	0.008
	0.3		0.002	0.006		0.3			0.001	0.006
	0.5		0.001	0.005		0.5			0.002	0.005
	0.7		0.001	0.003		0.7			0.001	0.003
	0.9		0.000	0.001		0.9			0.000	0.001
	0.7	0.2	−0.001	0.003		0.7	0.5	0.2	0.001	0.003
	0.9		−0.001	0.001		0.9			0.001	0.001

The Additional file 3 provides the simulation code of bias and mean square error for common AC_1_ using R language.

### Confidence intervals for common AC_1_

We conducted a simulation study to evaluate the performances of the three confidence intervals presented in the previous section. The coverage rates of the 95% confidence interval were examined. The balanced and unbalanced conditions for *π*_*k*_ and the balanced condition for *n*_*k*_ are considered. The results are shown in Table 5. The coverage rate of the SA method was generally lower than 0.95 under many conditions, with the exception of the value being close to 0.99 in the case of *n*_1_ = *n*_2_ = 20 and *γ*_0_= 0.9. The FZ method and PV method greatly improved the coverage rates close to the nominal level. However, the coverage rate of the PV method was closer to the nominal level than that of the FZ method in most cases under the conditions examined. The coverage rates of each method were also evaluated in the case of *K* = 3, and the unbalanced *n*_*k*_ conditions and both the FZ method and the PV method achieved coverage rates near 0.95 (results not shown).

**Table 5:** Coverage rates of common *γ* 95% confidence intervals of the three proposed methods based on 10,000 simulations

Balanced *π* conditions						Unbalanced *π* conditions						
*n*_1_ = *n*_2_	*γ*_0_	π_1_ = π_2_	SA	FZ	PV	*n*_1_ = *n*_2_	*γ*_0_	π_1_	π_2_	SA	FZ	PV
20	0.1	0.5	0.939	0.959	0.958	20	0.1	0.5	0.35	0.939	0.958	0.958
	0.3		0.936	0.958	0.950		0.3			0.933	0.958	0.955
	0.5		0.931	0.962	0.962		0.5			0.927	0.959	0.960
	0.7		0.924	0.976	0.963		0.7			0.917	0.971	0.961
	0.9		0.998	0.963	0.955		0.9			0.997	0.966	0.955
	0.1	0.35	0.935	0.953	0.956		0.1	0.65	0.35	0.938	0.955	0.957
	0.3		0.934	0.955	0.953		0.3			0.934	0.955	0.956
	0.5		0.926	0.956	0.955		0.5			0.927	0.959	0.955
	0.7		0.918	0.965	0.958		0.7			0.917	0.967	0.960
	0.9		0.996	0.967	0.947		0.9			0.996	0.969	0.950
	0.7	0.2	0.929	0.959	0.950		0.7	0.5	0.2	0.931	0.965	0.956
	0.9		0.970	0.966	0.911		0.9			0.993	0.970	0.943
50	0.1	0.5	0.949	0.953	0.952	50	0.1	0.5	0.35	0.946	0.952	0.953
	0.3		0.945	0.955	0.954		0.3			0.943	0.952	0.951
	0.5		0.945	0.954	0.953		0.5			0.941	0.952	0.952
	0.7		0.936	0.961	0.953		0.7			0.936	0.956	0.953
	0.9		0.920	0.971	0.971		0.9			0.923	0.968	0.965
	0.1	0.35	0.942	0.948	0.952		0.1	0.65	0.35	0.946	0.954	0.954
	0.3		0.942	0.950	0.950		0.3			0.943	0.953	0.952
	0.5		0.940	0.951	0.951		0.5			0.941	0.954	0.953
	0.7		0.937	0.954	0.952		0.7			0.936	0.956	0.953
	0.9		0.926	0.966	0.960		0.9			0.925	0.968	0.960
	0.7	0.2	0.938	0.949	0.948		0.7	0.5	0.2	0.937	0.954	0.952
	0.9		0.927	0.965	0.954		0.9			0.928	0.969	0.960
80	0.1	0.5	0.945	0.952	0.952	80	0.1	0.5	0.35	0.946	0.951	0.951
	0.3		0.947	0.952	0.952		0.3			0.946	0.952	0.952
	0.5		0.943	0.953	0.952		0.5			0.942	0.950	0.950
	0.7		0.944	0.950	0.949		0.7			0.943	0.955	0.953
	0.9		0.901	0.962	0.956		0.9			0.922	0.963	0.957
	0.1	0.35	0.946	0.951	0.951		0.1	0.65	0.35	0.943	0.947	0.946
	0.3		0.944	0.949	0.949		0.3			0.945	0.950	0.950
	0.5		0.944	0.950	0.950		0.5			0.944	0.953	0.952
	0.7		0.941	0.950	0.949		0.7			0.939	0.953	0.952
	0.9		0.931	0.960	0.953		0.9			0.927	0.963	0.954
	0.7	0.2	0.944	0.950	0.949		0.7	0.5	0.2	0.942	0.955	0.954
	0.9		0.929	0.956	0.951		0.9			0.927	0.961	0.955

SA, FZ, and PV refer to 95% confidence intervals for common AC_1_ using the simple asymptotic, Fisher’s Z transformation, and profile variance methods, respectively

The Additional file 4 provides the simulation code of confidence intervals for common AC_1_ using R language.

### An example

As an example, we used data from a randomized clinical trial called the Silicon Study, which was conducted to investigate the effectiveness of silicone fluids versus gases in the management of proliferative vitreoretinopathy (PVR) by vitrectomy [45]. The PVR classification, determined at the baseline visit, defines the severity of the disease as a continuum of increasing pathology graded as C3, D1, D2 or D3. The presence or absence of retinal injury in the superior nasal cavity was evaluated clinically by the operating ophthalmic surgeon and photographically by an independent fundus photograph reading center [46].

The data and results are summarized in Table 6. For reference, the results of the homogeneity score test proposed by Nam for the intra-class *κ* are also provided [15]. The probabilities of agreement in each stratum were from 0.800 to 0.880 and not so different. However, the values of *κ* in each stratum were from 0.117 to 0.520 and were greatly different. This might be due to the prevalence effect caused by the small values of *π*. In contrast, the values of *γ* were 0.723 to 0.861 and did not differ greatly among strata.

**Table 6:** Agreement between ophthalmologist and reading center classifying superior nasal retinal breaks stratified by PVR grade

	PVR grade			
	C3	D1	D2	D3
Both (*x*_1_)	1	6	5	3
One (*x*_2_)	9	8	11	9
Neither (*x*_3_)	65	46	54	33
Total (*n*)	75	60	70	45
*π*	0.073	0.167	0.150	0.167
*p*_*a*_	0.880	0.867	0.843	0.800
*κ* (MLE)	0.117	0.520	0.384	0.280
AC_1_ (MLE)	0.861	0.815	0.789	0.723

The proposed homogeneity score statistic $T\left({\overset{\sim }{\gamma}}_0,\overset{\sim }{\boldsymbol{\pi}}\right)$ was 2.060 (*p*-value = 0.560) and the homogeneity hypothesis was not rejected. The estimate of common AC_1_ was 0.808 and its 95% confidence intervals were 0.743–0.873 (SA method), 0.732–0.864 (FZ method) and 0.730–0.862 (PV method). Also, the score statistic for testing the homogeneity of *κ’s* [15] was 2.700 (*p*-value = 0.440) and the common *κ* was 0.352.

The Additional file 5 provides the code for clinical data examples using R language.

To investigate the sensitivity of the indicators to *π*_*k*_, we hypothetically considered more balanced and less balanced *π*_*k*_ under fixed *p*_*a*_ and *n*_*k*_ in each stratum. The generated data set and analysis results are summarized as Table S3 in the Additional file 1. *κ* was more sensitive to changes in the value of *π*, but AC_1_ was less sensitive to changes in the value of *π* than *κ*. The common AC_1_ was not affected as much as the common *κ* even if the *π* balance was lost.

## Discussion

It is well known that Cohen’s *κ* depends strongly on the marginal distributions, and Gwet proposed alternative and more stable measures of agreement, AC_1_ for nominal data and its extended agreement AC_2_ for ordinal data [24, 25]. A number of alternative measures have also been proposed, as in Holley and Guilford’s G [19], Aickin’s α [20], Andres and Marzo’s delta [21] and Marasini’s s* [22, 23]. Gwet [24] and Shankar and Bangdiwala [28] compared some measures and showed that AC_1_ has better properties than other kappa-type measures. In addition, AC_1_ has been utilized in the field of medical research over the past decade [29–35]. However statistical inference procedures of AC_1_ have not been discussed sufficiently. Therefore, Ohyama expressed AC_1_ using population parameters to develop a likelihood-based inference procedure and constructed confidence intervals of AC_1_ based on profile variances and likelihood ratios. Inclusion of subjects’ covariates, hypothesis testing and sample size estimation were also presented [36]. In the present study, the case of stratified data was discussed as one development of Ohyama [36] for two raters with binary outcomes. Furthermore, tests were derived for the homogeneity of AC_1_ between *K* independent strata and the inference of common AC_1_ was discussed.

In the numerical evaluation of type I error, both tests were conservative when the sample size was small and *γ*_0_ was 0.9, but the conservativeness was relaxed when the sample size was as large as 80. In other settings of simulation, the score test performed well while GOF sometimes could not achieve the nominal level. Therefore, we recommend using the score test for testing the homogeneity of AC_1_ among *K* strata. Note that, when zero cells are observed, the homogeneity score test statistic cannot be calculated. In such cases in our simulation study, we simply added 0.5 to the data set, which had no serious effect on the performance of the proposed score test in our simulation settings.

If the homogeneity assumption is reasonable, it may be desired to provide an estimate of the common AC_1_ as a summary measure of reliability. In the present study, we proposed an estimator of common AC_1_ and constructed its confidence intervals based on the SA, FZ, and PV methods. We also evaluated the performance of each numerically. The bias and MSE tended to be small as the sample size increased, and the results were nearly 0 when *n* = 80. The PV method provides coverage levels close to nominal in most situations, while the SA method tends to provide a shortage of coverage and the FZ method tends to provide excess coverage in some situations. Therefore, we recommend the PV method for calculating confidence intervals.

As in the PVR example, AC_1_ in each stratum is less affected by the prevalence or marginal probability than by the *κ*. It is suggested that the proposed homogeneity test and the general framework of common AC_1_ estimation are also essentially more stable than those of the *κ*.

There were some limitations in this study. First, as described above, the performance of the proposed score test was very conservative when *γ*_0_ = 0.9 and sample size was small. An exact approach might be an alternative method in such cases.

Next, in this study, the cases were limited to two raters with binary outcomes in each stratum. However, in the evaluation of medical data, it is often the case that multiple raters classify subjects into nominal or ordered categories. Our proposed method may be extended to the case of multiple raters with binary outcomes using the likelihood function for multiple raters. In the cases of two raters with nominal outcomes, Agresti [47] proposed a quasi-symmetry model with kappa as a parameter, and this technique may be extended to AC_1_ in the case of stratified data.

Finally, continuous covariates need to be categorized adequately to apply the proposed approach. The regression model proposed by Ohyama [36] can be used to assess the effect of continuous covariates on AC_1_, but it is limited to the case of two raters with binary data. Nelson and Edwards [48] and Nelson, Mitani and Edwards [49] proposed a method for constructing a measure of agreement using generalized linear mixed-effect models by introducing continuous latent variables representing the subject’s true disease status and for flexibly incorporating rater and subject covariates. These approaches might be applicable to AC_1_ and AC_2_.

## Conclusion

The method proposed in this study is considered to be useful for summarizing evaluations of consistency performed in multiple or stratified inter-rater agreement studies. In addition, the proposed method can be applied not only to medical or epidemiological research but also to assessment of the degree of consistency of characteristics, such as biometrics, psychological measurements, and data in the behavioral sciences.

### Supplementary information

**Additional file 1.** Supplementary tables.

**Additional file 2.** R code for type I errors and power.

**Additional file 3.** R code for Bias and MSE.

**Additional file 4.** R code for coverage rates.

**Additional file 5.** R code for clinical data examples.

## Data Availability

The program codes are shared in additional files. Clinical data referred to are from Barlow, et al. ref. [45].

## Appendix

### First and second derivatives of the log-likelihood function

The first and second derivatives of *l*(***γ***, ***π***), ***γ =*** (*γ*_1_, ∙ ∙ ∙, *γ*_*K*_)^′^***, π =*** (*π*_1_, ∙ ∙ ∙, *π*_*K*_)′ with respect to *r*_*k*_ and *π*_*k*_ are obtained as follows:

$$ {\displaystyle \begin{array}{l}\frac{\partial l}{\partial {\gamma}_k}=\frac{1}{2}{A}_k{R}_k,\\ {}\frac{\partial l}{\partial {\pi}_k}=\frac{x_{1k}}{P_{1k}}-\frac{x_{3k}}{P_{3k}}+\left(1-{\gamma}_k\right)\left(1-2{\pi}_k\right){R}_k,\\ {}\frac{\partial^2l}{\partial {\gamma}_k^2}=-\frac{1}{4}{A}_k^2{S}_k,\\ {}\frac{\partial^2l}{\partial {\gamma}_k\partial {\pi}_k}=-\frac{A_k}{2}\left[\frac{x_{1k}}{P_{1k}^2}-\frac{x_{3k}}{P_{3k}^2}+\left(1-{\gamma}_k\right)\left(1-2{\pi}_k\right){S}_k\right]-\left(1-2{\pi}_k\right){R}_k,\\ {}\frac{\partial^2l}{\partial {\pi}_k^2}=-\frac{x_{1k}}{P_{1k}^2}-\frac{x_{3k}}{P_{3k}^2}-2\left(\frac{x_{1k}}{P_{1k}^2}-\frac{x_{3k}}{P_{3k}^2}\right)\left(1-{\gamma}_k\right)\left(1-2{\pi}_k\right)-2\left(1-{\gamma}_k\right){R}_k-{\left(1-{\gamma}_k\right)}^2{\left(1-2{\pi}_k\right)}^2{S}_k,\\ {}\frac{\partial^2l}{\partial {\gamma}_k\partial {\gamma}_{k\hbox{'}}}=\frac{\partial^2l}{\partial {\pi}_k\partial {\pi}_{k\hbox{'}}}=\frac{\partial^2l}{\partial {\gamma}_k\partial {\pi}_{k\hbox{'}}}=0\kern0.50em \left(k\ne {k}^{\prime}\right),\end{array}} $$

where

$$ {\displaystyle \begin{array}{l}{A}_k=1-2{\pi}_k\left(1-{\pi}_k\right),\\ {}{R}_k=\frac{x_{1k}}{P_{1k}}-\frac{2{x}_{2k}}{P_{2k}}+\frac{x_{3k}}{P_{3k}},\\ {}{S}_k=\frac{x_{1k}}{P_{1k}^2}+\frac{4{x}_{2k}}{P_{2k}^2}+\frac{x_{3k}}{P_{3k}^2}.\end{array}} $$

Since *E*(*x*_*hk*_) = *n*_*k*_*P*_*hk*_(*γ*_*k*_) (*h* = 1, 2, 3),

$$ {\displaystyle \begin{array}{l}E\left(-\frac{\partial^2l}{\partial {\gamma}_k^2}\right)=\frac{n_k}{4}{A}_k^2{B}_k,\\ {}E\left(-\frac{\partial^2l}{\partial {\gamma}_k\partial {\pi}_k}\right)=\frac{n_k}{2}{A}_k{C}_k,\\ {}E\left(-\frac{\partial^2l}{\partial {\pi}_k^2}\right)={n}_k{D}_k,\end{array}} $$

where

$$ {\displaystyle \begin{array}{l}{B}_k=\frac{1}{P_{1k}}+\frac{4}{P_{2k}}+\frac{1}{P_{3k}},\\ {}{C}_k=\frac{1}{P_{1k}}-\frac{1}{P_{3k}}+\left(1-{\gamma}_k\right)\left(1-2{\pi}_k\right){B}_k,\\ {}{D}_k=\frac{1}{P_{1k}}+\frac{1}{P_{3k}}+\left(1-{\gamma}_k\right)\left(1-2{\pi}_k\right)\left(\frac{1}{P_{1k}}-\frac{1}{P_{3k}}+{C}_k\right).\end{array}} $$

Thus, the Fisher information matrix is given as follows:

$$ I\left(\boldsymbol{\gamma}, \boldsymbol{\pi} \right)=\frac{1}{4}\left[\begin{array}{cc}\operatorname{diag}\left({n}_k{A}_k^2{B}_k\right)& \operatorname{diag}\left(2{n}_k{A}_k{C}_k\right)\\ {}\operatorname{diag}\left(2{n}_k{A}_k{C}_k\right)& \operatorname{diag}\left(4{n}_k{D}_k\right)\end{array}\right]. $$

### Score test statistic for the null hypothesis

Define the score function *U* as

$$ U\left(\boldsymbol{\gamma}, \boldsymbol{\pi} \right)={\left(\partial l/\partial {\gamma}_1,\bullet \bullet \bullet, \partial l/\partial {\gamma}_K,\partial l/\partial {\pi}_1,\bullet \bullet \bullet, \partial l/\partial {\pi}_K\right)}^{\prime } $$

The score statistic for testing the null hypothesis *H*_0_ : *γ*_1_ = ⋯ = *γ*_*K*_ = *γ*_0_ is asymptotically distributed as a *χ*^2^ with *K*-1 degrees of freedom, and then expressed as

$$ T\left({\overset{\sim }{\gamma}}_0,\overset{\sim }{\boldsymbol{\pi}}\right)=U{\left({\overset{\sim }{\gamma}}_0,\overset{\sim }{\boldsymbol{\pi}}\right)}^{\prime }I{\left({\overset{\sim }{\gamma}}_0,\overset{\sim }{\boldsymbol{\pi}}\right)}^{-1}U\left({\overset{\sim }{\gamma}}_0,\overset{\sim }{\boldsymbol{\pi}}\right), $$

where ${\overset{\sim }{\gamma}}_0$ and ${\overset{\sim }{\pi}}_k$ are the maximum likelihood estimators under *H*_0_, and then score function vector is expressed as $U\left({\overset{\sim }{\gamma}}_0,\overset{\sim }{\boldsymbol{\pi}}\right)=\frac{1}{2}{\left({\overset{\sim }{A}}_1{\overset{\sim }{R}}_1,\dots, {\overset{\sim }{A}}_K{\overset{\sim }{R}}_K,0,\dots, 0\right)}^{\prime }.$

The upper left K × K matrix of $I{\left(\overset{\sim }{\boldsymbol{\gamma}},\overset{\sim }{\boldsymbol{\pi}}\right)}^{-1}$ is expressed as follows:

$$ {\displaystyle \begin{array}{l}{I}^{\gamma \gamma}\left({\overset{\sim }{\gamma}}_0,\overset{\sim }{\boldsymbol{\pi}}\right)=4{\left(\operatorname{diag}\left({n}_k{\overset{\sim }{A}}_k^2{\overset{\sim }{B}}_k\right)-\operatorname{diag}\left(2{n}_k{\overset{\sim }{A}}_k{\overset{\sim }{C}}_k\right){\operatorname{diag}}^{-1}\left(4{n}_k{\overset{\sim }{D}}_k\right)\operatorname{diag}\left(2{n}_k{\overset{\sim }{A}}_k{\overset{\sim }{C}}_k\right)\right)}^{-1}\\ {}=4\operatorname{diag}\left[\frac{{\overset{\sim }{D}}_k}{n_k{\overset{\sim }{A}}_k^2\left({\overset{\sim }{B}}_k{\overset{\sim }{D}}_k-{\overset{\sim }{C}}_k^2\right)}\right],\end{array}} $$

so that, the score test statistic Eq. (11) is derived.

### Confidence interval for *γ*_0_ based on Fisher’s Z transformation

Fisher’s Z transformation of ${\overset{\sim }{\gamma}}_0$ is defined by

$$ \overset{\sim }{z}=\frac{1}{2}\log \frac{1+{\overset{\sim }{\gamma}}_0}{1-{\overset{\sim }{\gamma}}_0}. $$

Using the delta method, the asymptotic variance of $\overset{\sim }{z}$ is represented by

$$ Var\left(\overset{\sim }{z}\right)={\left(\frac{1}{1-{\gamma}_0^2}\right)}^2 Var\left({\overset{\sim }{\gamma}}_0\right), $$

where $Var\left(\overset{\sim }{\gamma}\right)$ is given by Eq. (14). Then a confidence interval for *z* = 0.5 log[(1 + *γ*_0_)/(1 − *γ*_0_)] is obtained by

$$ {C}_{\pm }=\overset{\sim }{z}\pm {Z}_{\alpha /2}\sqrt{\hat{Var}\left(\overset{\sim }{z}\right)}, $$

where $\hat{Var}\left(\overset{\sim }{z}\right)$ is defined by substituting ${\overset{\sim }{\gamma}}_0$ and ${\overset{\sim }{\pi}}_k$ into $Var\left(\overset{\sim }{z}\right)$. Thus the confidence interval for *γ*_0_ based on FZ method is obtained as follows:

$$ \left(\frac{\exp \left(2{C}_{-}\right)-1}{\exp \left(2{C}_{-}\right)+1},\frac{\exp \left(2{C}_{+}\right)-1}{\exp \left(2{C}_{+}\right)+1}\right). $$

### Derivation of Eq. (14)

By the second-order partial derivatives of the log-likelihood function *l*_0_(*γ*_0_, ***π***) and taking expectations, we obtain

$$ {\displaystyle \begin{array}{l}{I}_{\gamma \gamma}=E\left(-\frac{\partial^2{l}_0}{\partial {\gamma_0}^2}\right)=\frac{1}{4}\sum \limits_{k=1}^K{n}_k{A}_k^2{B}_k^{(0)},\\ {}{I}_{kk}=E\left(-\frac{\partial^2{l}_0}{\partial {\pi}_k^2}\right)={n}_k{D}_k^{(0)},\\ {}{I}_{\gamma k}=E\left(-\frac{\partial^2{l}_0}{\partial {\gamma}_0\partial {\pi}_k}\right)=\frac{1}{2}{n}_k{A}_k{C}_k^{(0)},\end{array}} $$

where ${B}_k^{(0)},{C}_k^{(0)}$ and ${D}_k^{(0)}$ are values using *γ*_*k*_ = *γ*_0_ in *B*_*k*_, *C*_*k*_ and *D*_*k*_ respectively. Let

$$ I=\left(\begin{array}{cc}{I}_{\gamma \gamma}& {I}_{\gamma \pi}\\ {}{I}_{\gamma \pi}^{\prime }& {I}_{\pi \pi}\end{array}\right), $$

where *I*_*γπ*_ = (*I*_*γ*1_, …, *I*_*γK*_) and *I*_*ππ*_ = diag(*I*_*kk*_). When *P*_1*k*_ ≠ 0, *P*_2*k*_ ≠ 0 and *P*_3*k*_ ≠ 0 for all *k*, *I*_*ππ*_ is non-singular matrix and then the element corresponding to *I*_*γγ*_ of the inverse matrix of *I*, which is the variance of ${\overset{\sim }{\gamma}}_0$, is given by

$$ {I}^{\gamma \gamma}={\left({I}_{\gamma \gamma}-{I}_{\gamma \pi}{I}_{\pi \pi}^{-1}{I}_{\gamma \pi}^{\prime}\right)}^{-1}={\left({I}_{\gamma \gamma}-\sum \limits_{k=1}^K{I}_{\gamma k}^2{I}_{kk}^{-1}\right)}^{-1}=4{\left[\sum \limits_{k=1}^K{n}_k{A_k}^2\left({B}_k^{(0)}-\frac{{C_k^{(0)}}^2}{D_k^{(0)}}\right)\right]}^{-1}. $$

Since

$$ \frac{4{D}_k^{(0)}}{B_k^{(0)}{D}_k^{(0)}-{C}_k^{(0)2}}=\left(1-{p}_{a,k}\right){p}_{a,k}+\left(1-{\gamma}_0\right)\left(1-2{p}_{e,k}^{\ast}\right)\left(1+{p}_{a,k}\right), $$

where ${p}_{a,k}={\gamma}_0\left(1-{p}_{e,k}^{\ast}\right)+{p}_{e,k}^{\ast }$ is the probability of agreement in the *k*-th stratum and ${p}_{e,k}^{\ast }=2{\pi}_k\left(1-{\pi}_k\right)$ is the probability of chance agreement in the *k*-th stratum, and using the variance formula for the single stratum case given by Ohyama [36], *I*^*γγ*^ can be reduced to the rightmost expression in Eq. (14).

### Profile variance approach

The profile variance of a statistic is defined as the variance similar to the estimated variance but without substituting the estimate for the parameter corresponding to the statistic [41].

In this study, ${\overset{\sim }{\pi}}_k$ is substituted for *π*_*k*_ in Eq. (15), and then we obtain the profile variance $\overset{\sim }{Var}\left({\overset{\sim }{\gamma}}_0\right)$ from (14). Since ${\overset{\sim }{\gamma}}_0$ is distributed asymptotically as the normal distribution with mean *γ*_0_ and variance (14), we have

$$ \frac{{\left({\overset{\sim }{\gamma}}_0-{\gamma}_0\right)}^2}{\overset{\sim }{Var}\left({\overset{\sim }{\gamma}}_0\right)}\to {\chi}_1^2. $$

Thus we can obtain the confidence limits as the two admissible roots of Eq. (17).

Since Eq. (17) is a cubic equation for *γ*_0_ and is complicated to solve, thus we calculated the confidence limit by numerical calculation. The program is given in additional file. Other examples of the profile variance approach for obtaining confidence intervals can be found in many literatures. For example, Bickel and Doksum [50] reported a confidence interval based on the profile variance for the one-sample binomial proportion. Rothman [51] and Afifi, Elashoff and Lee JJ [52] described profile variance type of confidence intervals for survival probability.

## Abbreviations

FZ: Fisher’s Z transformation
GOF: goodness-of-fit
MLE: maximum likelihood estimator
MSE: mean squared error
PV: profile variance
PVR: proliferative vitreoretinopathy
SA: simple asymptotic
