# Supplementary material for: Homogeneity score test of AC1 statistics and estimation of common AC1 in multiple or stratified inter-rater agreement studies
Source: BMC Med Res Methodol. 2020 Feb 5;20:20. doi: 10.1186/s12874-019-0887-5 (PMC7001312; doi:10.1186/s12874-019-0887-5)
Supplement: Supplementary file 1 — Additional file 1. Supplementary tables. [file 12874_2019_887_MOESM1_ESM.docx]

Additional file 1

Supplemental Tables for “Homogeneity score test of AC_1_ statistics and estimation of common AC_1_ in multiple or stratified inter-rater agreement studies” by Chikara Honda and Tetsuji Ohyama.

Empirical type I error rate for the homogeneity test

The results when *K* = 3 are shown in Table S1. The type I error rate of SCORE is conservative, as in the case of *K* = 2, to less than 0.01 when n = 20 and $\gamma=$ 0.9, but it is almost maintained at the nominal level in other cases. It also tends to be closer to the nominal level as the sample size increases. In contrast, the performance of GOF is unsatisfactory in most cases.

Table S2 shows the results for the case when *K* = 3 and sample sizes are unbalanced among strata. The type I error rate of SCORE does not greatly exceed the nominal level, but the type I error rate of GOF is inflated at the nominal level in many situations.

Sensitivity analysis

For Barlow Lai and Azen 1991 data (Table 6), numerical values of $p_{a}$ and *n* of each strata were fixed, and more/less balanced data for $\pi$ was prepared. Specifically, the More balanced data is obtained by modifying the numerical values of the C3 stratum, and the less balanced data is obtained by modifying the numerical values of the D2 and D3 strata.The generated data set and analysis results are shown in the Table S3.

As a result, *κ* is sensitive to changes in the value of $\pi$, but AC_1_ was less sensitive to changes in the value of $\pi$ than *κ*. The common AC_1_ was not affected as much as the common *κ* even if the $\pi$ balance was lost.

Table S1

Empirical type I error rates of homogeneity tests for $\gamma_{1}=\gamma_{2}=\gamma_{3}=\gamma$ based on 10,000 simulations (*K =* 3 balanced sample size)

| Balanced π conditions | | | | |  | Unbalanced π conditions | | | | | | |
| --- | --- | --- | --- | --- | --- | --- | --- | --- | --- | --- | --- | --- |
| n_1_ = n_2_ = n_3_ = 20 | | | |  |  | n_1_ = n_2_ = n_3_ = 20 | | | | | |  |
|  | γ | π_1_ = π_2_ = π_3_ | SCORE | GOF |  |  | γ | π_1_ | π_2_ | π_3_ | SCORE | GOF |
|  | 0.1 | 0.5 | 0.040 | 0.076 |  |  | 0.1 | 0.5 | 0.35 | 0.65 | 0.043 | 0.129 |
|  | 0.3 |  | 0.042 | 0.066 |  |  | 0.3 |  |  |  | 0.046 | 0.121 |
|  | 0.5 |  | 0.045 | 0.064 |  |  | 0.5 |  |  |  | 0.047 | 0.100 |
|  | 0.7 |  | 0.030 | 0.040 |  |  | 0.7 |  |  |  | 0.033 | 0.058 |
|  | 0.9 |  | 0.004 | 0.006 |  |  | 0.9 |  |  |  | 0.005 | 0.008 |
|  | 0.1 | 0.35 | 0.048 | 0.155 |  |  | 0.1 | 0.65 | 0.35 | 0.5 | 0.044 | 0.131 |
|  | 0.3 |  | 0.051 | 0.152 |  |  | 0.3 |  |  |  | 0.047 | 0.124 |
|  | 0.5 |  | 0.051 | 0.118 |  |  | 0.5 |  |  |  | 0.048 | 0.103 |
|  | 0.7 |  | 0.037 | 0.071 |  |  | 0.7 |  |  |  | 0.032 | 0.059 |
|  | 0.9 |  | 0.006 | 0.009 |  |  | 0.9 |  |  |  | 0.004 | 0.008 |
|  | 0.7 | 0.2 | 0.043 | 0.156 |  |  | 0.7 | 0.5 | 0.2 | 0.65 | 0.035 | 0.092 |
|  | 0.9 |  | 0.010 | 0.033 |  |  | 0.9 |  |  |  | 0.006 | 0.015 |
| n_1_ = n_2_ = n_3_ = 50 | | |  |  |  | n_1_ = n_2_ = n_3_ = 50 | | | | | | |
|  | 0.1 | 0.5 | 0.044 | 0.057 |  |  | 0.1 | 0.5 | 0.35 | 0.65 | 0.046 | 0.169 |
|  | 0.3 |  | 0.048 | 0.056 |  |  | 0.3 |  |  |  | 0.049 | 0.121 |
|  | 0.5 |  | 0.050 | 0.056 |  |  | 0.5 |  |  |  | 0.051 | 0.090 |
|  | 0.7 |  | 0.048 | 0.053 |  |  | 0.7 |  |  |  | 0.049 | 0.067 |
|  | 0.9 |  | 0.024 | 0.025 |  |  | 0.9 |  |  |  | 0.024 | 0.028 |
|  | 0.1 | 0.35 | 0.051 | 0.221 |  |  | 0.1 | 0.65 | 0.35 | 0.5 | 0.047 | 0.169 |
|  | 0.3 |  | 0.052 | 0.158 |  |  | 0.3 |  |  |  | 0.048 | 0.124 |
|  | 0.5 |  | 0.052 | 0.108 |  |  | 0.5 |  |  |  | 0.053 | 0.094 |
|  | 0.7 |  | 0.053 | 0.076 |  |  | 0.7 |  |  |  | 0.052 | 0.070 |
|  | 0.9 |  | 0.027 | 0.032 |  |  | 0.9 |  |  |  | 0.024 | 0.030 |
|  | 0.7 | 0.2 | 0.052 | 0.202 |  |  | 0.7 | 0.5 | 0.2 | 0.65 | 0.049 | 0.110 |
|  | 0.9 |  | 0.035 | 0.065 |  |  | 0.9 |  |  |  | 0.029 | 0.039 |
| n_1_ = n_2_ = n_3_ = 80 | | |  |  |  | n_1_ = n_2_ = n_3_ = 80 | | | | | | |
|  | 0.1 | 0.5 | 0.047 | 0.054 |  |  | 0.1 | 0.5 | 0.35 | 0.65 | 0.049 | 0.181 |
|  | 0.3 |  | 0.044 | 0.051 |  |  | 0.3 |  |  |  | 0.046 | 0.117 |
|  | 0.5 |  | 0.053 | 0.057 |  |  | 0.5 |  |  |  | 0.054 | 0.088 |
|  | 0.7 |  | 0.048 | 0.050 |  |  | 0.7 |  |  |  | 0.050 | 0.068 |
|  | 0.9 |  | 0.037 | 0.038 |  |  | 0.9 |  |  |  | 0.042 | 0.047 |
|  | 0.1 | 0.35 | 0.049 | 0.238 |  |  | 0.1 | 0.65 | 0.35 | 0.5 | 0.054 | 0.180 |
|  | 0.3 |  | 0.052 | 0.153 |  |  | 0.3 |  |  |  | 0.050 | 0.121 |
|  | 0.5 |  | 0.053 | 0.103 |  |  | 0.5 |  |  |  | 0.053 | 0.091 |
|  | 0.7 |  | 0.052 | 0.074 |  |  | 0.7 |  |  |  | 0.053 | 0.070 |
|  | 0.9 |  | 0.042 | 0.048 |  |  | 0.9 |  |  |  | 0.040 | 0.045 |
|  | 0.7 | 0.2 | 0.051 | 0.205 |  |  | 0.7 | 0.5 | 0.2 | 0.65 | 0.049 | 0.112 |
|  | 0.9 |  | 0.046 | 0.076 |  |  | 0.9 |  |  |  | 0.043 | 0.056 |

Table S2

Empirical type I error rates of homogeneity tests for $\gamma_{1}=\gamma_{2}=\gamma_{3}=\gamma$ based on 10,000 simulations (*K* = 3 unbalanced sample size) n_1_ = 20, n_2_ = 50, n_3_ = 80

| Balanced π conditions | | | |  | Unbalanced π conditions | | | | | |
| --- | --- | --- | --- | --- | --- | --- | --- | --- | --- | --- |
| γ | π_1_ = π_2_ = π_3_ | SCORE | GOF |  | γ | π_1_ | π_2_ | π_3_ | SCORE | GOF |
| 0.1 | 0.5 | 0.042 | 0.065 |  | 0.1 | 0.5 | 0.35 | 0.65 | 0.050 | 0.173 |
| 0.3 |  | 0.043 | 0.059 |  | 0.3 |  |  |  | 0.044 | 0.118 |
| 0.5 |  | 0.044 | 0.055 |  | 0.5 |  |  |  | 0.048 | 0.085 |
| 0.7 |  | 0.040 | 0.046 |  | 0.7 |  |  |  | 0.040 | 0.059 |
| 0.9 |  | 0.029 | 0.031 |  | 0.9 |  |  |  | 0.027 | 0.032 |
| 0.1 | 0.35 | 0.049 | 0.193 |  | 0.1 | 0.65 | 0.35 | 0.5 | 0.046 | 0.153 |
| 0.3 |  | 0.053 | 0.154 |  | 0.3 |  |  |  | 0.047 | 0.127 |
| 0.5 |  | 0.050 | 0.107 |  | 0.5 |  |  |  | 0.048 | 0.101 |
| 0.7 |  | 0.046 | 0.073 |  | 0.7 |  |  |  | 0.045 | 0.069 |
| 0.9 |  | 0.033 | 0.039 |  | 0.9 |  |  |  | 0.029 | 0.035 |
| 0.7 | 0.2 | 0.047 | 0.190 |  | 0.7 | 0.5 | 0.2 | 0.65 | 0.042 | 0.101 |
| 0.9 |  | 0.038 | 0.065 |  | 0.9 |  |  |  | 0.030 | 0.042 |

Table S3

Sensitivity analysis

Agreement between ophthalmologist and reading center classifying superior nasal retinal breaks stratified by PVR grade

| PVR grade / original data | | |  |  |  | artificial PVR grade / more balanced data | | | | |  | artificial PVR grade / less balanced data | | | | |
| --- | --- | --- | --- | --- | --- | --- | --- | --- | --- | --- | --- | --- | --- | --- | --- | --- |
|  | C3 | D1 | D2 | D3 |  |  | C3 | D1 | D2 | D3 |  |  | C3 | D1 | D2 | D3 |
| Both (*x*_1_) | 1 | 6 | 5 | 3 |  | Both (*x*_1_) | 8 | 6 | 5 | 3 |  | Both (*x*_1_) | 1 | 6 | 10 | 18 |
| One (*x*_2_) | 9 | 8 | 11 | 9 |  | One (*x*_2_) | 9 | 8 | 11 | 9 |  | One (*x*_2_) | 9 | 8 | 11 | 9 |
| Neither (*x*_3_) | 65 | 46 | 54 | 33 |  | Neither (*x*_3_) | 58 | 46 | 54 | 33 |  | Neither (*x*_3_) | 65 | 46 | 49 | 18 |
| Total (*n*) | 75 | 60 | 70 | 45 |  | Total (*n*) | 75 | 60 | 70 | 45 |  | Total (*n*) | 75 | 60 | 70 | 45 |
| *π* | 0.073 | 0.167 | 0.150 | 0.167 |  | *π* | 0.167 | 0.167 | 0.150 | 0.167 |  | *π* | 0.073 | 0.167 | 0.221 | 0.500 |
| *p*_a_ | 0.880 | 0.867 | 0.843 | 0.800 |  | *p*_a_ | 0.880 | 0.867 | 0.843 | 0.800 |  | *p*_a_ | 0.880 | 0.867 | 0.843 | 0.800 |
| *κ* (MLE) | 0.117 | 0.520 | 0.384 | 0.280 |  | *κ* (MLE) | 0.568 | 0.520 | 0.384 | 0.280 |  | *κ* (MLE) | 0.117 | 0.520 | 0.544 | 0.600 |
| AC_1_ (MLE) | 0.861 | 0.815 | 0.789 | 0.723 |  | AC_1_ (MLE) | 0.834 | 0.815 | 0.789 | 0.723 |  | AC_1_ (MLE) | 0.861 | 0.815 | 0.760 | 0.600 |

| common *κ* test |  |  |  | common *κ* test |  |  |  | common *κ* test |  |  |
| --- | --- | --- | --- | --- | --- | --- | --- | --- | --- | --- |
| statistic | df | p-value |  | statistic | df | p-value |  | statistic | df | p-value |
| 2.70 | 3 | 0.440 |  | 2.05 | 3 | 0.562 |  | 4.10 | 3 | 0.251 |
| common *κ* |  |  |  | common *κ* |  |  |  | common *κ* |  |  |
| 0.352 |  |  |  | 0.454 |  |  |  | 0.497 |  |  |
| common AC_1_ test | |  |  | common AC_1_ test | |  |  | common AC_1_ test | |  |
| statistic | df | p-value |  | statistic | df | p-value |  | statistic | df | p-value |
| 2.06 | 3 | 0.560 |  | 1.192 | 3 | 0.755 |  | 5.908 | 3 | 0.116 |
| common AC_1_ | 95% CI (PV method) | |  | common AC_1_ | 95% CI (PV method) | |  | common AC_1_ | 95% CI (PV method) | |
| 0.808 | 0.730 - 0.862 | |  | 0.797 | 0.715 - 0.854 | |  | 0.784 | 0.699- 0.844 | |
